# To Dr. Bradley

**Published:** 1809-03

**Authors:** 


### To Dr. BRADLEY.

SIR,

IN the Medical and Physical Journal for last month, there is a Paper upon the Treatment of Hydrophobia, by Dr. Wood, for which he is entitled to much praise. As an addition to his remedies, might not a large portion of cantharides mixed with his plaster, be made use of with a prospect of advantage?

P. S. Might not large doses of æther be serviceable?

*Kingston, February* 12, 1809.

